# Improvement of Yield and Brewing Quality of Brewing Wheat Grown Under Reduced Nitrogen Fertilizer and Exogenous Melatonin Application

**DOI:** 10.3390/foods15183335

**Published:** 2026-09-20

**Authors:** Jianchao Feng, Zifei Ge, Beiming Xu, Chenchen Cao, Wenhua Ma, Geng Ma, Yingxin Xie, Dongyun Ma

**Affiliations:** 1 College of Agronomy and National Engineering Research Center for Wheat, Henan Agricultural University, Zhengzhou 450046, China; 2 National Wheat Technology Innovation Center, Henan Agricultural University, Zhengzhou 450046, China; 3 Tobacco Monopoly Bureau of Luoshan, Xinyang 464200, China; 4 State Key Laboratory of High-Efficiency Production of Wheat-Maize Double Cropping, Zhengzhou 450046, China

**Keywords:** exogenous melatonin, nitrogen reduction, brewing wheat, yield, quality

## Abstract

This study aimed to investigate the effects of exogenous melatonin on grain yield and quality of brewing wheat grown under reduced nitrogen (N) fertilizer application. Field experiments were performed using two brewing wheat cultivars, Yunong 901 and Fanmai 8. Treatments included conventional N (225 kg∙ha^−1^) and reduced N (150 kg∙ha^−1^ and 120 kg∙ha^−1^) levels, each with melatonin or water spray. The results demonstrated that reducing N fertilizer application led to decreased net photosynthetic rate, specific leaf N, and photosynthetic N use efficiency, while exogenous melatonin application improved corresponding photosynthetic parameters in all three N treatments. Reducing N decreased grain yield and grain endogenous hormone levels. Exogenous melatonin application increased grain yield under all N levels, with the highest increases in YN901 (10.61%) under MN and FM8 (10.27%) under LN. Melatonin partially restored grain hormone levels, particularly in the late filling stage under LN, maintaining higher filling activity. While reducing N decreased liquor volatile flavor compounds (alcohols, esters, N compounds), melatonin application significantly increased the total ester content and the relative levels of multiple volatile flavor compounds. Melatonin mitigated the adverse effects of N reduction, improving photosynthesis, grain yield, and liquor flavor quality. Comprehensively, reducing N to 150 kg∙ha^−1^ combined with exogenous melatonin application improves grain yield, N efficiency, and liquor flavor quality.

## 1. Introduction

Baijiu, a traditional Chinese distilled spirit, occupies a unique position in Chinese culture [1]. As a popular alcoholic beverage with distinctive flavor and quality, it is highly valued by consumers [2,3,4]. Baijiu plays a vital role in China’s food and drink industry [5]. According to the National Bureau of Statistics, the number of large-scale baijiu enterprises and annual production reached 1040 and 7.407 billion liters, respectively, in 2020. Concurrently, the industry’s total sales revenue reached CNY 583.639 billion, with an annual profit of CNY 158.541 billion [6]. The saying “the aroma of liquor arises from its grains” emphasizes raw materials as one of the key factors influencing fermentation quality [7,8]. In Chinese baijiu production, wheat functions not only as a raw material for daqu (starter culture) [9] but also as a primary fermentation grain [10,11]. Wheat accounts for more than 15% of the raw materials required for strong-flavor baijiu [11]. With rising living standards, the demand for baijiu has increased, driving greater need for wheat as a production input. Therefore, improving the grain yield and quality of brewing wheat remains critical for premium baijiu production.

Nitrogen (N) fertilizer application is a crucial agronomic practice in wheat production, which significantly regulates grain yield and the formation of nutritional components, particularly proteins and starch. Starch constitutes the primary component of brewing wheat, and content in raw materials partially determines liquor quality [12]. Liquor yield is influenced not only by the total starch content but also by the amylose-to-amylopectin ratio [10]. Protein, the second-largest nutrient in wheat, critically affects fermentation, flavor compound formation, and liquor yield. During fermentation, N compounds produce diverse flavor compounds and modulate fermentation performance and aroma profiles [13,14]. Thus, N application affects brewing quality by altering grain nutritional composition. Early research has demonstrated that N application rates significantly affect total acid and total ester contents in liquor: the highest total acid content and lowest fusel alcohol content occurred at 90 kg∙ha^−1^ N, while the total ester content was lowest at 135 kg∙ha^−1^ N [10]. Research has reported that the sensory evaluation score of daqu peaked at 180 kg∙ha^−1^ N [15]. Excessive N application not only compromises grain yield and quality but also reduces photosynthetic nitrogen use efficiency (PNUE), increases production costs, and causes environmental issues [16].

Melatonin, also known as *N*-acetyl-5-methoxytryptamine, is a naturally occurring indoleamine small-molecule compound. Initially identified in higher plants in 1993 [17], melatonin has since been detected in numerous plant species [18]. Melatonin is distributed throughout plant organs, where it facilitates essential growth-promoting effects through its potent antioxidant activity and activation of MAPK cascades [19]. Melatonin has emerged as a bioactive molecule with diverse regulatory capabilities, demonstrating significant roles in seed germination under stress conditions and plant growth regulation [20]. In addition, melatonin exhibits notable effects on crop yield and quality enhancement. Previous research has demonstrated that 100 µmol∙L^−1^ or 1 mmol∙L^−1^ melatonin applications enhanced strawberry fruit color, firmness, and total soluble solids content [21]. Research has shown that 100 µmol∙L^−1^ melatonin significantly improved grain-filling characteristics and starch physicochemical properties in soft wheat [22]. It has also been established that melatonin regulates panicle architecture in rice by modulating hormone levels, thereby increasing yield while enhancing processing and nutritional quality [23]. Foliar melatonin application also increased kernels per ear, thousand kernel weight (TKW), and grain yield in maize [24]. However, most previous studies have focused on the individual effects of either N reduction or exogenous melatonin application on crop productivity and quality, with limited attention given to their combined effects on brewing wheat. In particular, the interactive mechanisms by which melatonin supplementation mitigates *N*-deficiency-induced constraints on photosynthetic performance, endogenous hormone dynamics, and grain filling, while simultaneously modulating the volatile flavor compound profile of baijiu, remain largely unexplored. Therefore, examining the effects of exogenous melatonin application on growth, grain yield, and brewing quality of brewing wheat grown under reduced N regimes would provide valuable insights into high-quality and environmentally sustainable production.

## 2. Materials and Methods

### 2.1. Materials and Experimental Design

The experiment was conducted during the 2023–2024 growing season at two sites: Henan Agricultural University Experimental Station, Zhengzhou, Henan Province, China (34.87° N, 113.60° E) and Xinxiang Science and Education Park of Henan Agricultural University, Henan Province, China (35.6° N, 113.56° E). Soil at both sites was classified as fluvo-aquic soil, with the following properties in the 0–20 cm tillage layer: organic matter 17.52 and 13.93 g∙kg^−1^, total N 0.91 and 0.81 g∙kg^−1^, available phosphorus 17.84 and 13.85 mg∙kg^−1^, and available potassium 209.67 and 154.87 mg∙kg^−1^ for Zhengzhou and Xinxiang sites, respectively. Detailed meteorological data, including precipitation and mean temperature during the growing seasons, are presented in Figure S1. A split–split-plot design was used: N treatments including conventional N level (225 kg∙ha^−1^ or high N [HN]) and reduced N levels (150 kg∙ha^−1^ or moderate [MN] and 120 kg∙ha^−1^ or low [LN]) were set as the main plots; two wheat cultivars, Yunong 901 (YN901) and Fanmai 8 (FM8), were set as subplots; and exogenous melatonin foliar spray (100 μmol∙L^−1^ melatonin [T1]) and water-sprayed control (T0) were set as sub-subplots. YN901 is a new wheat cultivar specifically bred for brewing, jointly developed by Henan Agricultural University (Zhengzhou, Henan, China) and China Kweichow Moutai Distillery Co., Ltd. (Renhuai, Guizhou, China), and approved by the Henan Provincial Crop Variety Approval Committee in June 2021, while FM8 is a high-yielding and stable-yielding wheat cultivar bred by the Agricultural Science Institute of Dishen Seed Industry in the Huangfanqu Area of Henan Province, and is one of the preferred cultivars for brewing and Qu-making enterprises. Urea (≥46.0% N; Henan Xinlianxin Chemicals Group Co., Ltd., Xinxiang, Henan, China), granular superphosphate (≥12% available P_2_O_5_; Anhui Fengle Fertilizer Co., Ltd., Hefei, China), and potassium sulfate (≥52% water-soluble K_2_O; Henan Xinlianxin Chemicals Group Co., Ltd., Xinxiang, China) were used as the N, P, and K fertilizer sources, respectively. Phosphorus (P_2_O_5_) and potassium (K_2_O) fertilizers were applied basally at 150 kg∙ha^−1^ each. Nitrogen was split-applied: 50% basal + 50% top-dressed at the jointing stage. Plot size was 6 m^2^ (3 m × 2 m), with three replicates. Sowing dates were 16 October 2023 in Zhengzhou and 17 October 2023 in Xinxiang. Wheat was mechanically sown in rows at a seeding rate of 150 kg·ha^−1^, with a row spacing of 20 cm and a sowing depth of 3–5 cm. Irrigation was applied at the jointing and flowering stages at a rate of 750 m^3^·ha^−1^ per irrigation. Melatonin application was initiated at the full heading stage; 45 mL∙m^−2^ sprayed daily at 16:00, every 5 days, totaling three applications. Control plots received water spray. Other management followed local high-yielding practices.

### 2.2. Physiological Index Analysis

Flag leaves with similar growth patterns were selected for physiological index analysis. The chlorophyll relative content (soil plant analysis development; SPAD) and leaf N content were measured using a plant nutrient meter (TYS-4N; Zhejiangtuopu Instrument Co., Ltd., Hangzhou, China), while leaf area was determined using a portable laser leaf area meter (CI-202; CID Bio-Science, Camas, WA, USA). Net photosynthetic rate (Pn) was measured using a LI-6800 portable photosynthesis and fluorescence system (LI-COR Biosciences, Lincoln, NE, USA) between 09:00 and 11:00 on clear days. The middle portion of fully expanded, well-illuminated flag leaves with similar growth status was selected, and 3–5 plants were measured for each treatment. During measurements, the leaf chamber conditions were controlled at a photosynthetically active radiation (PAR) of 1000 μmol·m^−2^·s^−1^, a reference CO_2_ concentration of 400 μmol·mol^−1^, a leaf chamber temperature of 25 °C, and a flow rate of 500 μmol·s^−1^. Data were recorded after Pn and other gas-exchange parameters had stabilized for approximately 2–3 min. Leaf mass per area, specific leaf N (SLN), and photosynthetic nitrogen use efficiency (PNUE) were calculated according to Pinto et al. [25].

### 2.3. Endogenous Hormone Content Determination

The contents of indole-3-acetic acid (IAA), abscisic acid (ABA), gibberellic acid (GA_3_), and melatonin in wheat grains were determined using enzyme-linked immunosorbent assay kits and reagents from Shanghai Enzyme-Linked Biotechnology Co., Ltd. (Shanghai, China), according to the instructions provided with the assay kits.

### 2.4. Starch and Protein Content Determination

The total starch and amylose contents of wheat flour were analyzed by the method of He et al. [26]. The amylopectin content was calculated by subtracting the amylose content from the total starch content. The protein content was measured using a near-infrared grain analyzer (DA7200; Perten Instruments, Huddinge, Sweden) according to the instrument’s operation instructions.

### 2.5. Evaluation of the Brewing Quality of Wheat Grains

**Figure 1:**
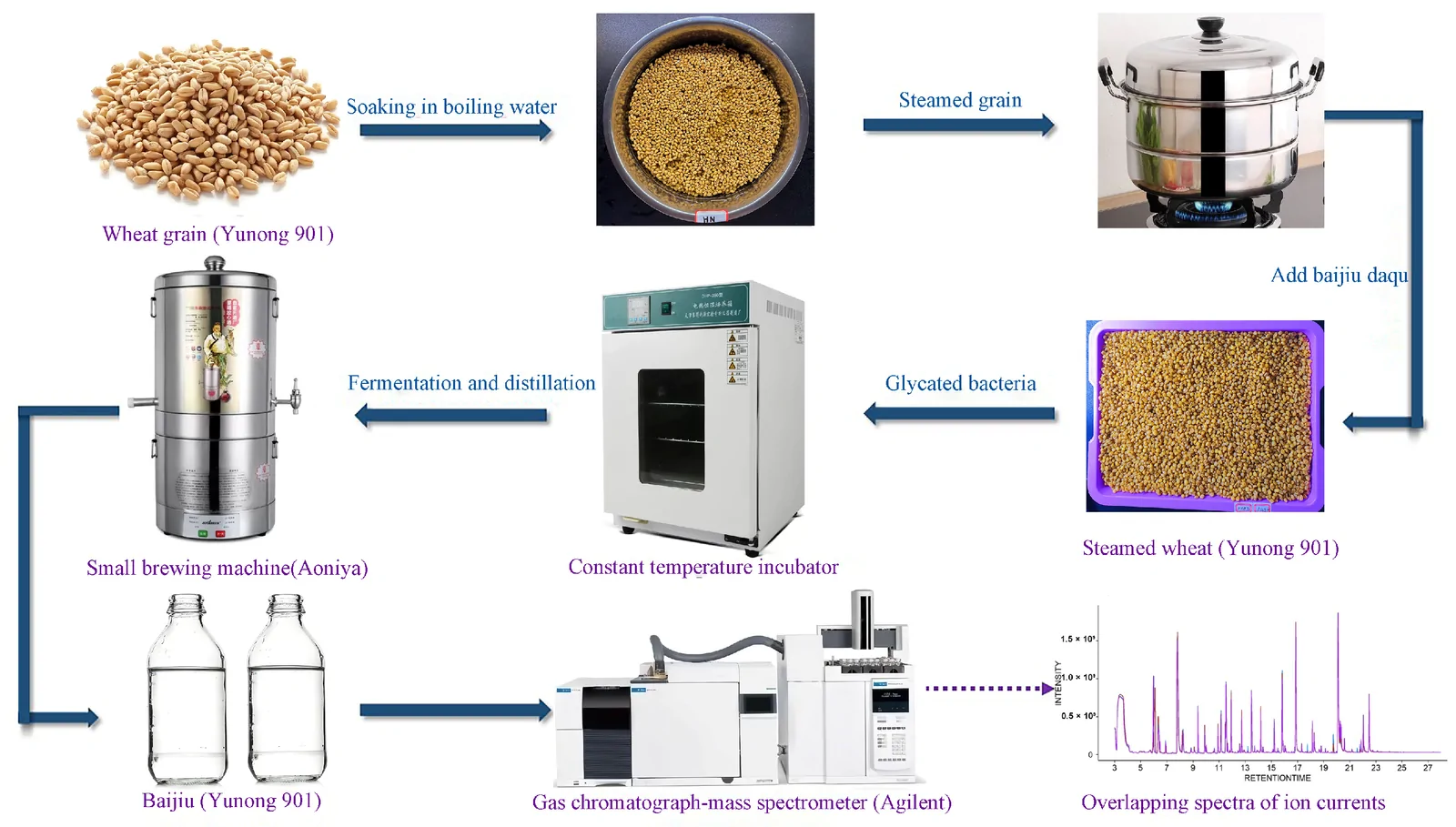
Flowchart of the Production of Brewed Liquor from Wheat.

Laboratory-scale liquor brewing was conducted using a fermentation–distillation integrated system (Model JB5A; Aoniya, Zhongshan, China) following the method of Liu et al. [27]. The procedure comprised the following steps (Figure 1): (1) Grain soaking: 800 g of wheat grains was soaked in 1 L distilled water for 24 h, followed by rinsing to remove impurities. (2) Steaming: Grains were steamed over boiling water until achieving a moderately firm texture with ≥85% grain splitting rate (approximately 2.5 h). (3) Starter addition: Steamed grains were spread evenly, cooled to 40 °C, and uniformly mixed with 2.8 g of jiuqu starter (starch-based liquor starter; Huaxi Distillery Co., Ltd., Pengzhou, China). (4) Saccharification and culturing: The mixture was covered with double-layered gauze and incubated at 30 °C for 48 h for saccharification and microbial cultivation. (5) Fermentation: The saccharified material was transferred to the integrated system, mixed with water (1:1 ratio), and fermented anaerobically at 30 °C for 14 days. (6) Distillation: Post-fermentation, the mash was distilled to collect raw liquor, with liquor yield recorded.

Ethanol concentration was measured using a precision alcohol meter (CJM-091; Chuangjimei Co., Ltd., Hengshui, China). Liquor yield was calculated based on the 60% (*v*/*v*) ethanol standard:

liquor yield (%) = (liquor output/raw grain input) × 100%

The total acid content was quantified by acid–base titration (Chinese National Standard GB/T 10345-2022), expressed as acetic acid equivalent. The total ester content was determined by saponification followed by acid–base titration (GB/T 10345-2022), reported as ethyl acetate equivalent. Fusel alcohols were measured following the method of Feng et al. (2024) [28].

### 2.6. Determination of Volatile Compounds in Liquor

Gas chromatography coupled with time-of-flight mass spectrometry (GC-TOF-MS) was used to analyze volatile compounds in liquor. Briefly, a 5-mL liquor sample was added to a 20-mL Agilent headspace bottle, then placed on the sampling tray and analyzed by GC-TOF-MS. The analysis was performed using an Agilent 7890B GC-TOF-MS system (Pegasus BT, Leco Corporation, St. Joseph, MI, USA). A DB-WAX capillary column (30 m × 0.25 mm × 0.25 μm) was used for separation. Helium was used as the carrier gas at a constant flow rate of 1.0 mL∙min^−1^. Injection temperature was set to 245 °C. The source temperature was 220 °C. The temperature was initially maintained at 40 °C for 3 min, then raised to 245 °C at a rate of 10 °C∙min^−1^. The energy was 70 eV in electron impact mode. MS data were acquired in the full-scan mode with the *m*/*z* range 35–450 at a rate of 15 spectra per second. Headspace solid-phase micro-extraction (HS-SPME) conditions: extraction head with DVB/CAR/PDMS 50/30 μm, 2 cm; extraction temperature of 80 °C; and incubation, extraction, and conditioning time of 10, 30, and 5 min, respectively. To ensure analytical quality, pooled quality control samples were analyzed alongside experimental samples.

The raw data were processed using ChromaTOF (v4.71; Leco) for automated baseline denoising and smoothing, peak picking, deconvolution, and peak alignment. Compounds were identified by matching the mass spectrum and retention index with the NIST database. After sum-normalization, the processed data underwent multivariate data analysis, including Pareto-scaled principal component analysis and orthogonal partial least-squares discriminant analysis (OPLS-DA), using the ropls R package in R (v4.3.2). The variable importance in projection (VIP) value of each variable in the OPLS-DA model was calculated to indicate its contribution to the classification. VIP > 1 and *p*-value < 0.05 were used to screen significant differentially expressed volatiles (DEVs).

### 2.7. Statistical Analysis

Data were analyzed by analysis of variance and Duncan’s multiple-range tests using SPSS v23.0 (IBM SPSS Statistics v22, SPSS Inc., Chicago, IL, USA). Statistical significance was set at *p* < 0.05.

## 3. Results

### 3.1. Photosynthetic Parameters of Flag Leaves in Brewing Wheat Grown Under Reduced N and Exogenous Melatonin Application

**Figure 2:**
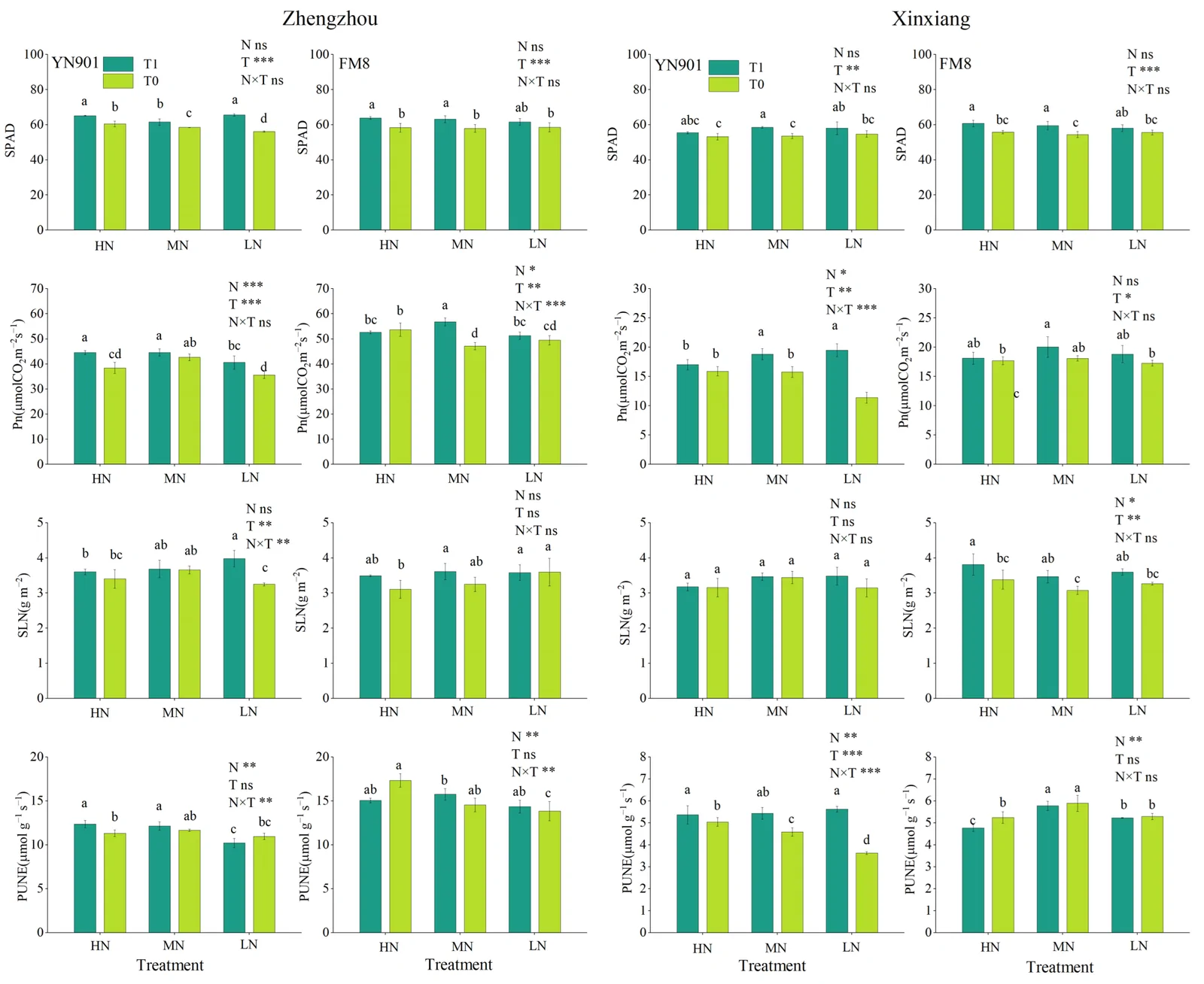
Effects of exogenous melatonin application on photosynthetic parameters of flag leaves in wheat grown under reduced nitrogen (N) fertilizer application. HN, MN, and LN represent high, medium, and low N application levels, respectively. T1 and T0 indicate melatonin and water spray (control), respectively. N and T indicate the main effects of nitrogen application level and melatonin treatment, respectively; N×T indicates their interaction. ns, not significant; *, **, and *** indicate *p* < 0.05, *p* < 0.01, and *p* < 0.001, respectively. Different lowercase letters above the column represent significant difference between treatments at the 0.05 level.

Flag leaf SPAD values of both wheat cultivars generally decreased under reduced N conditions, except for YN901 under LN treatment at the Zhengzhou site (Figure 2). Exogenous melatonin application increased flag leaf SPAD values across all N treatments. At the Zhengzhou site, SPAD values of YN901 under HN, MN, and LN conditions increased by 7.71%, 5.25%, and 16.86%, respectively, compared with controls, while FM8 increased by 9.61%, 9.03%, and 5.42%. At the Xinxiang site, SPAD values of YN901 and FM8 increased by 4.24–9.14% and 4.50–9.25%, respectively, following melatonin application. Pn values were higher under the HN condition than under the LN condition. For YN901, exogenous melatonin application enhanced flag leaf Pn in all N treatments: at Zhengzhou, Pn increased by 15.90% (HN), 4.56% (MN), and 13.93% (LN) compared with controls, while at Xinxiang, the increases were 7.06% (HN), 19.34% (MN), and 71.02% (LN). For FM8, melatonin application minimally affected Pn under HN but increased Pn by 20.53% and 10.79% under MN (Zhengzhou and Xinxiang sites), and by 3.72% and 8.94% under LN. N in leaves facilitates light reception and energy conversion, and PNUE indicates efficiency of N allocation in photosynthetic systems. The results indicate that melatonin application significantly enhanced PNUE for YN901 under LN and FM8 under MN, with average increases of 8.50% and 5.44% compared with controls (T0). Exogenous melatonin also increased the SLN content in all N treatments: YN901 showed average increases of 9.72% and 4.04%, while FM8 exhibited average increases of 7.76% and 11.84% at Zhengzhou and Xinxiang sites, respectively. These findings demonstrate that exogenous melatonin application enhances leaf photosynthetic characteristics under varying N regimes, with the strongest response exhibited by YN901 under LN and FM8 under MN.

### 3.2. Endogenous Hormone Content in Brewing Wheat Grown Under Reduced N and Exogenous Melatonin Application

**Figure 3:**
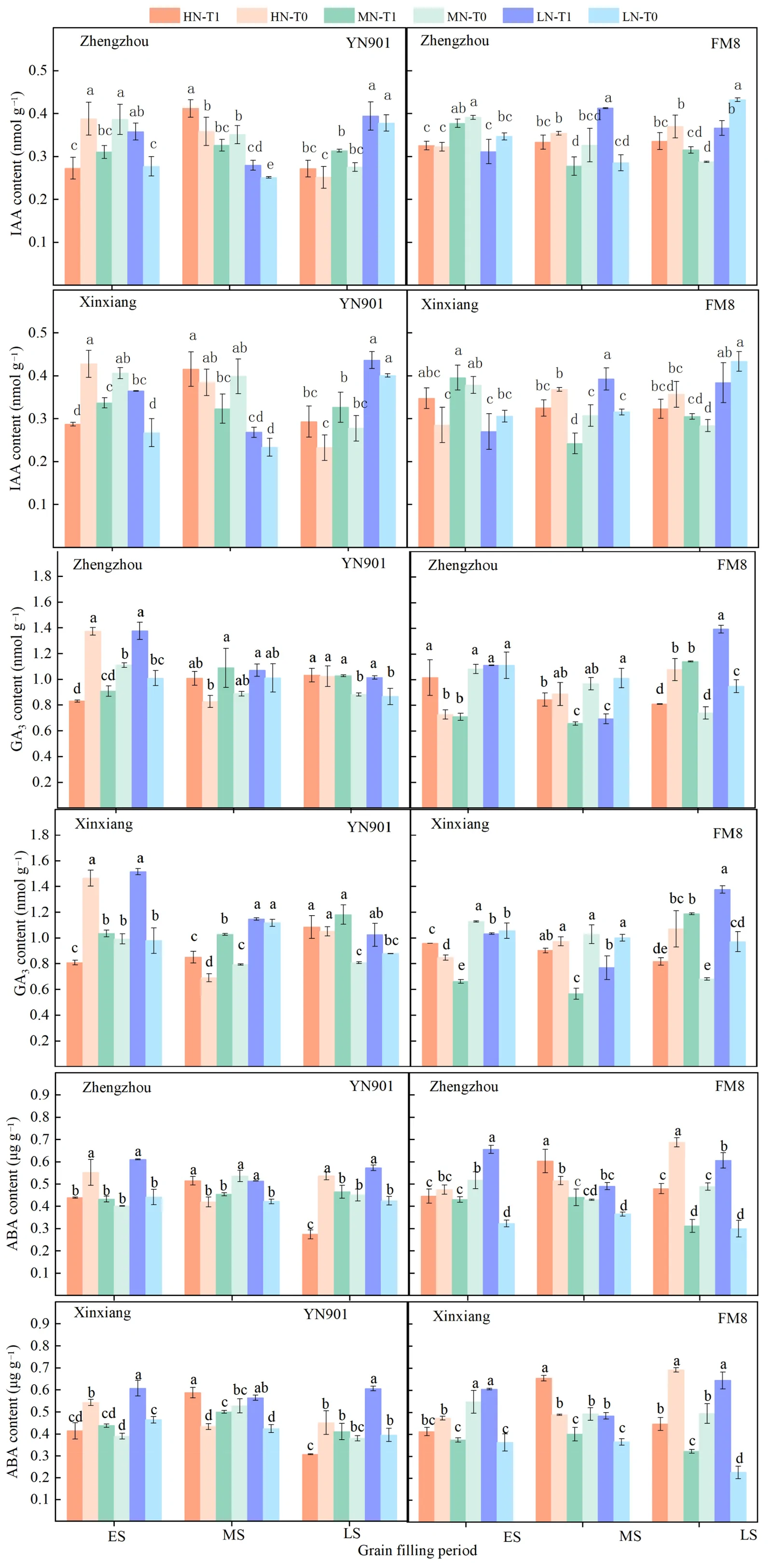
Effects of exogenous melatonin application on endogenous hormone contents of wheat grown under reduced N fertilizer application. ES, MS, and LS represent early, middle, and late grain-filling stages, respectively. Different lowercase letters above the bars indicate significant differences among treatments within the same grain-filling stage at *p* < 0.05.

Grain IAA content of YN901 exhibited a gradual decrease with N fertilizer reduction during the middle grain-filling stage but demonstrated an upward trend in later stages (Figure 3). Exogenous melatonin application differentially affected grain IAA in both cultivars across N regimes: under HN, melatonin application showed no significant difference or slight decreases relative to control (T0), whereas under LN, melatonin significantly elevated the grain IAA content, which increased by 12.21–15.69% throughout filling in YN901, and by 19.59% (Xinxiang) and 30.75% (Zhengzhou) at mid-filling in FM8. These findings indicate that exogenous melatonin application generally enhances grain IAA accumulation, particularly under LN conditions.

Exogenous melatonin significantly enhanced the GA_3_ content of YN901 grains during the middle and late filling stages across all N treatments (Figure 3), averaging 26.01% higher than control (T0) under MN. For FM8, melatonin application increased late-filling GA_3_ by 64.15% (MN) and 44.27% (LN) vs. control (T0), demonstrating that melatonin promotes GA_3_ accumulation during the late grain-filling stage under low-to-moderate N rates.

Grain ABA content of both cultivars demonstrated a general decrease with N reduction during the middle and late filling stages (Figure 3). Under LN treatment, exogenous melatonin significantly elevated ABA throughout grain development. For YN901, the ABA content increased by 35.56%, 27.39%, and 44.61% at the early, middle, and late filling stages compared with control (T0), respectively; while for FM8, the increases were 86.76%, 32.88%, and 135.85%, respectively. These results confirm that melatonin application generally enhances ABA levels across all grain-filling stages under N deficit, with the most pronounced effect during late filling.

### 3.3. Grain Yield of Brewing Wheat Grown Under Reduced N and Exogenous Melatonin Application

As N application levels decreased, spike numbers exhibited a declining trend, while the number of grains per spike and 1000-grain weight initially increased then decreased, reaching maximum values under MN treatment (Table 1). Grain yield decreased proportionally with reduced N fertilizer application. In all three N levels, exogenous melatonin spray showed no significant effect on spike number but consistently enhanced both number of grains per spike and 1000-grain weight. The increase in 1000-grain weight was particularly notable under the MN level, with average increases of 5.63% and 1.21% for the two cultivars compared with their respective controls. Regarding grain yield, YN901 demonstrated its largest increase (10.61%) under MN-T1 application, while FM8 exhibited its largest increase (10.27%) under LN-T1 application, compared with controls. The highest average grain yield for YN901 was observed under MN-T1 application, reaching 8387.04 kg∙ha^−1^, while FM8 achieved the maximum grain yield under the HN-T1 application at 8371.87 kg∙ha^−1^. Overall, grain weight and grain yield showed declining trends with reduced N application. However, foliar melatonin spray partially mitigated grain yield reduction caused by N reduction through increased number of grains per spike and 1000-grain weight.

### 3.4. Grain Starch and Protein Contents in Brewing Wheat Grown Under Reduced N and Exogenous Melatonin Application

With decreasing N fertilizer application levels, the total starch and amylopectin contents of both cultivars demonstrated an increasing trend (Table 2), reaching peak values under LN treatment. Compared with the HN level, YN901 showed increases of 1.50% total starch and 2.84% amylopectin, while FM8 exhibited increases of 2.26% total starch and 3.01% amylopectin. The amylose content of both cultivars initially increased then decreased with N reduction, reaching minimum levels under LN. Melatonin application enhanced the total starch content across all three N levels, with significant increases observed under the HN. The melatonin application notably reduced the amylose content compared with control (T0) under the MN level for both YN901 (average decrease: 10.14%) and FM8 (average decrease: 7.84%). Following melatonin application, the total starch content was 70.18–73.57% for YN901 and 71.12–73.43% for FM8. These findings indicate that reduced N fertilizer application enhanced grain total starch and amylopectin contents while reducing the amylose content. In addition, melatonin spray under different N levels consistently increased the total starch content. Both N reduction and exogenous melatonin application significantly improved grain total starch content, which is a critical quality parameter for brewing wheat. The protein content decreased with reduced N application, reaching minimum values under the LN level, averaging 11.85% for YN901 and 12.46% for FM8. Exogenous melatonin application produced no significant changes in the protein content of either cultivar at the Zhengzhou site. However, at the Xinxiang site, melatonin application significantly increased the protein content of both cultivars compared with control.

### 3.5. Brewing Characteristics of Wheat Grains and Volatile Compounds in Liquor

**Figure 4:**
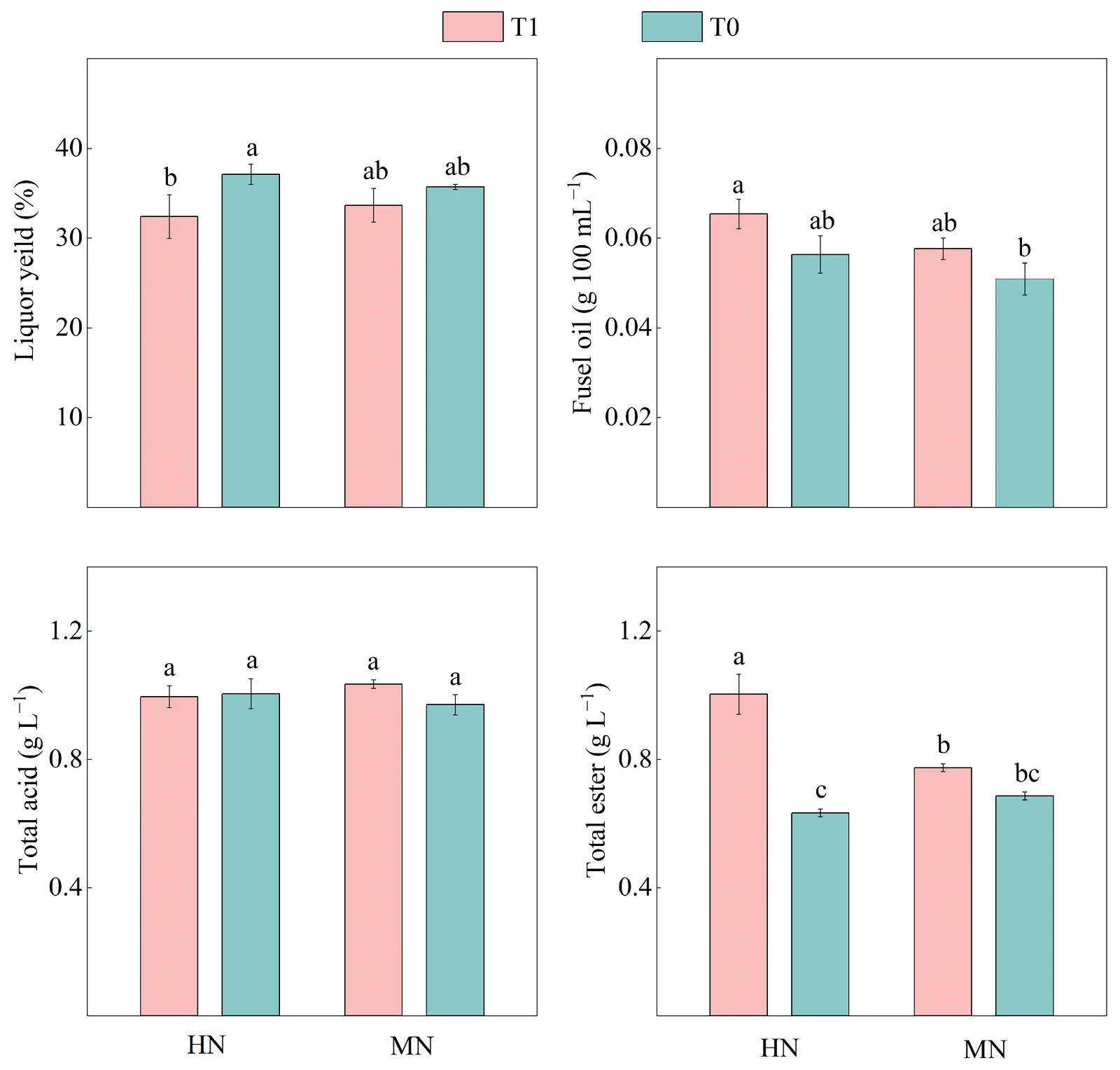
Brewing characteristics of wheat grown under reduced N fertilizer and exogenous melatonin application. HN and MN represent high and medium N application levels, respectively. T1 and T0 indicate melatonin and water spray (control), respectively. Different lowercase letters above the column represent significant difference between treatments at the 0.05 level.

To further examine the effect of exogenous melatonin on wheat grain brewing characteristics, small-scale brewing experiments were performed on YN901 grains under two N levels (HN and MN) and exogenous melatonin application. Decreased N application levels led to reduced total esters and fusel alcohols in the brewed liquor, while total acids and liquor yield remained relatively stable (Figure 4). Under different N fertilizer levels, melatonin spray decreased liquor yield but caused no significant changes in total acid and fusel alcohol contents. However, the total ester content significantly increased under both N levels, with average increases of 58.33% and 12.82% under HN and MN levels, respectively.

**Figure 5:**
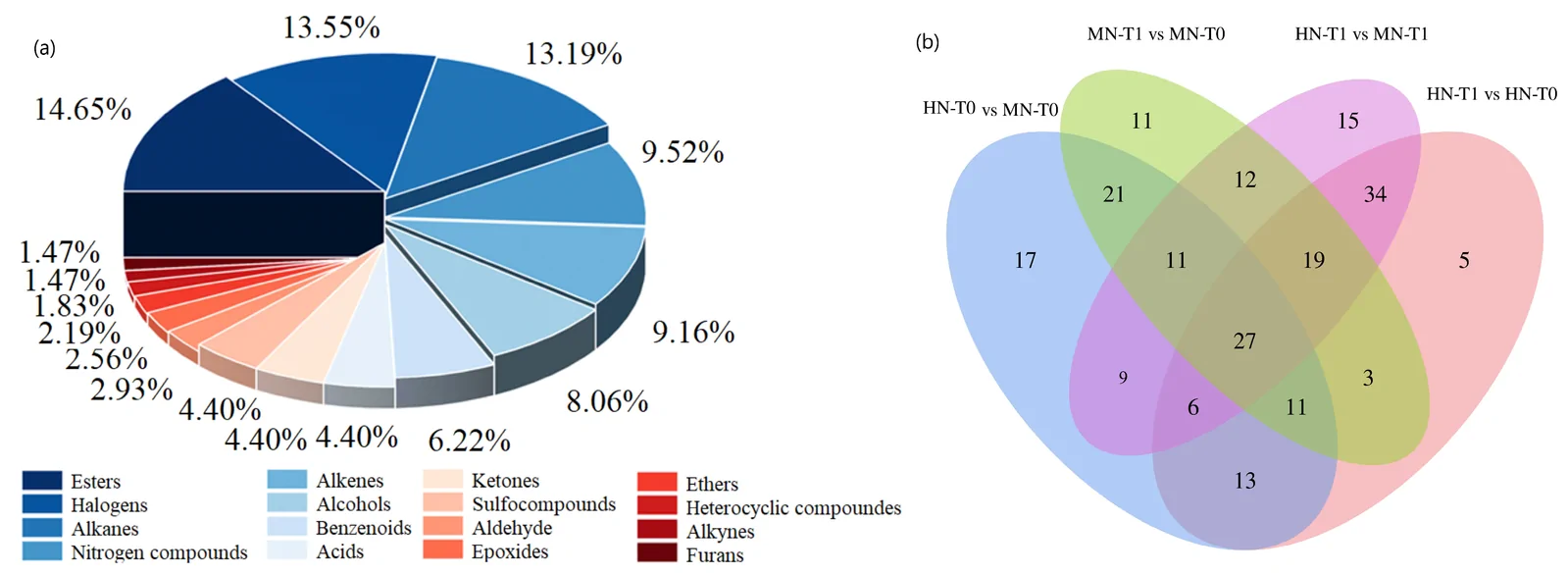
Classification of identified volatile flavor compounds (**a**) and Venn diagram of differentially expressed volatiles (DEVs) (**b**) in brewed liquor from wheat grown under HN and MN conditions with melatonin application.

Moreover, HS-SPME coupled with GC-MS (HS-SPME-GC-MS) was used to analyze the volatile flavor compounds in brewing liquor from YN901 wheat grains. A total of 273 volatile flavor compounds were identified and categorized into 16 classes (Figure 4, Table S1). These compounds included esters (14.65%), halogens (13.55%), alkanes (13.19%), N compounds (9.50%), alkenes (9.16%), alcohols (8.06%), acids (4.40%), and sulfur compounds (4.40%). In addition, 53 flavor compounds were common between HN-T0 vs. MN-T0 and HN-T1 vs. MN-T1, while 60 flavor compounds were common to HN-T1 vs. HN-T0 and MN-T1 vs. MN-T0 (Figure 5).

**Figure 6:**
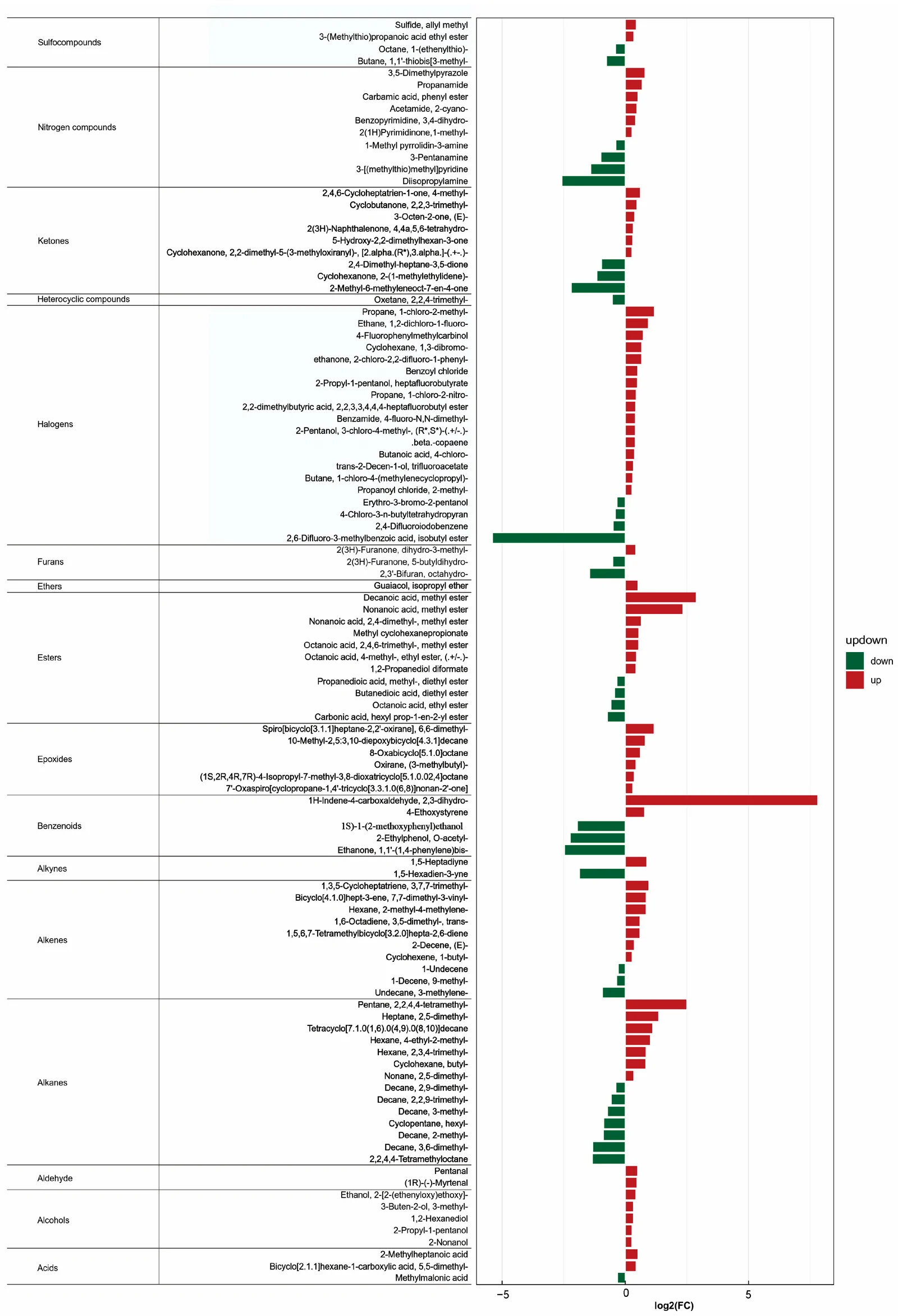
DEVs between HN and MN conditions without melatonin application.

A total of 106 significant DEVs were identified in HN-T0 vs. MN-T0 (VIP ≥ 1, foldchange ≥ 1.2 or foldchange ≤ 0.83 and *p*-value < 0.05), comprising 71 upregulated and 35 downregulated volatile compounds under the HN level (Figure 6, Table S2). Among these DEVs, the esters with the highest expression levels were 1,2-propanediol diformate and octanoic acid ethyl ester, with average relative levels of 8882.08 and 8005.65, respectively. The N compound with the highest expression level was 3-[(methylthio)methyl]-pyridine, with a relative average level of 30,366.06. Under HN treatment, all 5 differentially expressed alcohols were upregulated, while 7 of 11 differentially expressed esters were upregulated and 4 were downregulated. Decanoic acid methyl ester (contributing floral and fruity flavor to baijiu) increased 7.38-fold under HN treatment, while octanoic acid ethyl ester (imparting a fruity odor) increased under MN treatment. Under HN-T0 treatment, the level of 1H-indene-2,3-dihydro-4-carboxaldehyde was 224.43-fold higher than under MN treatment, while the levels of 2,6-difluoro-3-methylbenzoic acid isobutyl ester and diisopropylamine (characterized by a stimulating ammonia odor) decreased.

**Figure 7:**
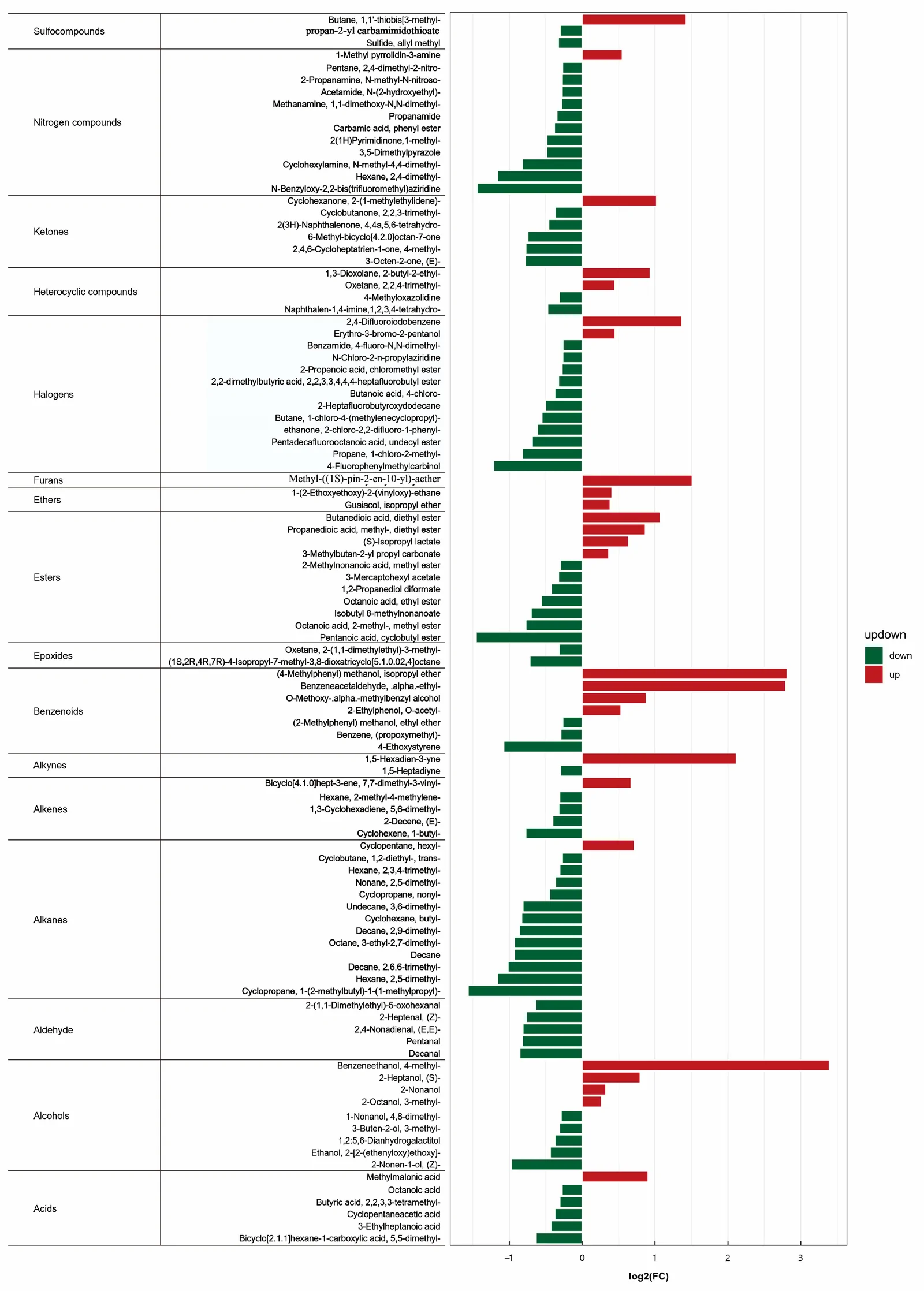
DEVs identified in T1 and T0 under HN condition.

In comparison with control (T0) under the HN condition, 101 DEVs were identified following melatonin application, including 26 upregulated and 75 downregulated compounds (Table S3). Among the six differentially expressed acids, five were downregulated and only 1 was upregulated (Figure 7). Of the nine differentially expressed alcohols, four were upregulated and five were downregulated. Notably, 4-methyl-benzeneethanol (associated with rose fragrance) showed a 10.49-fold increase under melatonin application. Among the 11 differentially expressed esters, four were upregulated and seven were downregulated. Specifically, (S)-isopropyl lactate (contributing wine yogurt flavor) and butanedioic acid diethyl ester (contributing cooked apple flavor) showed 1.56-fold and 2.10-fold upregulation, respectively. Thirteen DEVs belonging to the halogen class were identified between HN-T1 and HN-T0 treatments. Only two were upregulated in T1, while 11 were downregulated. These findings indicate that exogenous melatonin application modifies the flavor compound profile in grain-brewed baijiu while reducing the halogenated compound content.

**Figure 8:**
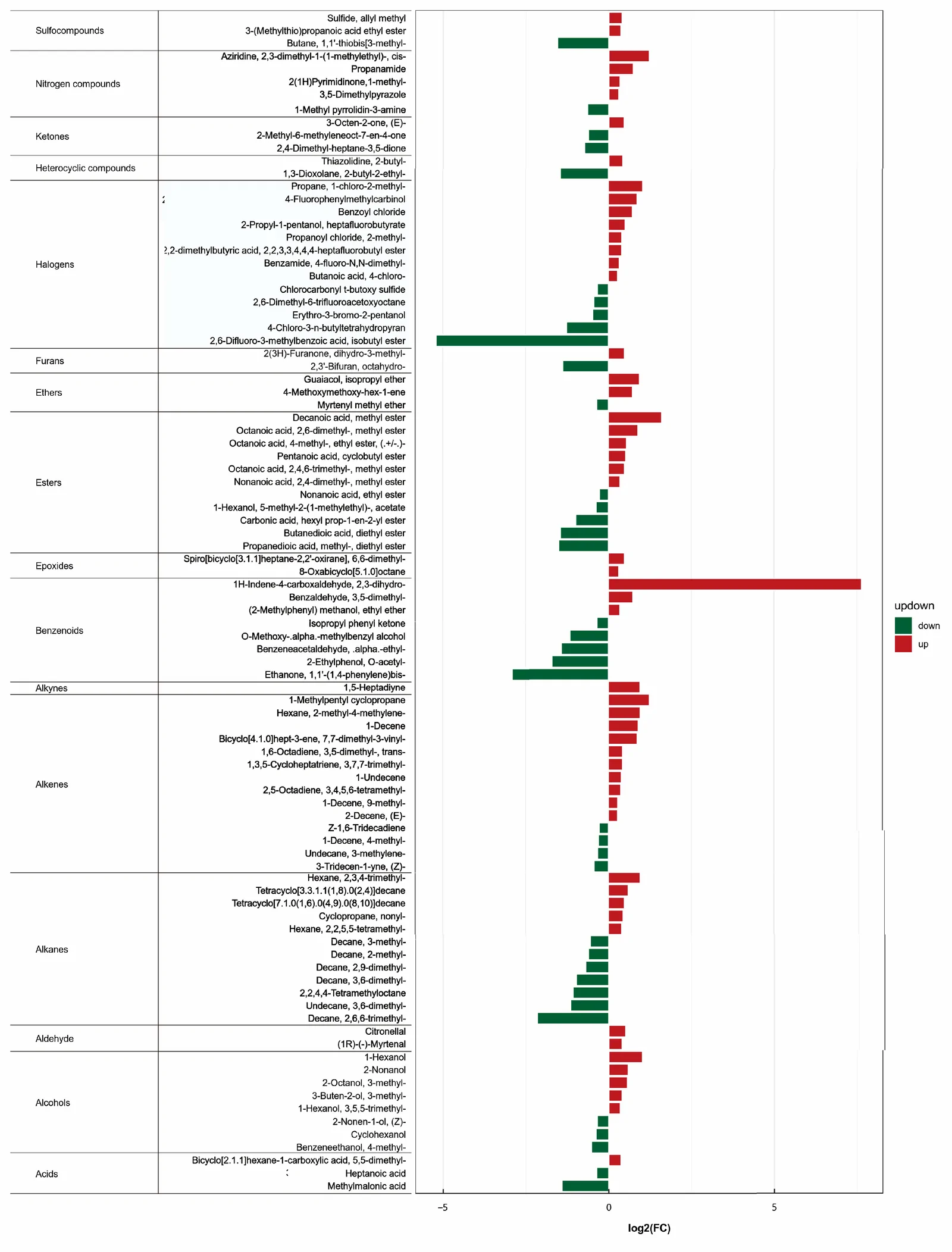
DEVs identified in T1 and T0 under the MN condition.

Compared with control (T0) under the MN condition, exogenous melatonin application yielded 92 DEVs in T1; of these, 54 were upregulated and 38 were downregulated (Figure 8, Table S4). Among the eight differentially expressed alcohols, five demonstrated increased levels. Specifically, 1-hexanol and 2-nonanol (associated with green fruity odor) increased by 2.02-fold and 1.50-fold, respectively. Eleven esters showed differential levels; melatonin application increased the levels of 6 esters, with decanoic acid methyl ester showing 3.05-fold upregulation. 3-(Methylthio)propanoic acid ethyl ester (contributing pineapple fruity notes) and allyl methyl sulfide (contributing alliaceous savory notes) increased by 1.29-fold and 1.32-fold, respectively. The two differentially expressed aldehydes, (1R)-(−)-myrtenal and citronellal, increased by 1.32-fold and 1.42-fold, respectively. Conversely, the halogenated compound 2,6-difluoro-3-methylbenzoic acid isobutyl ester showed a significantly decreased level after melatonin application. These results demonstrate that under MN conditions, exogenous melatonin treatment increases the flavor compounds in grain-brewed liquors like baijiu, particularly alcohols, esters, alkenes, aldehydes, and N and sulfur compounds.

### 3.6. Correlation and Redundancy Analysis (RDA)

**Figure 9:**
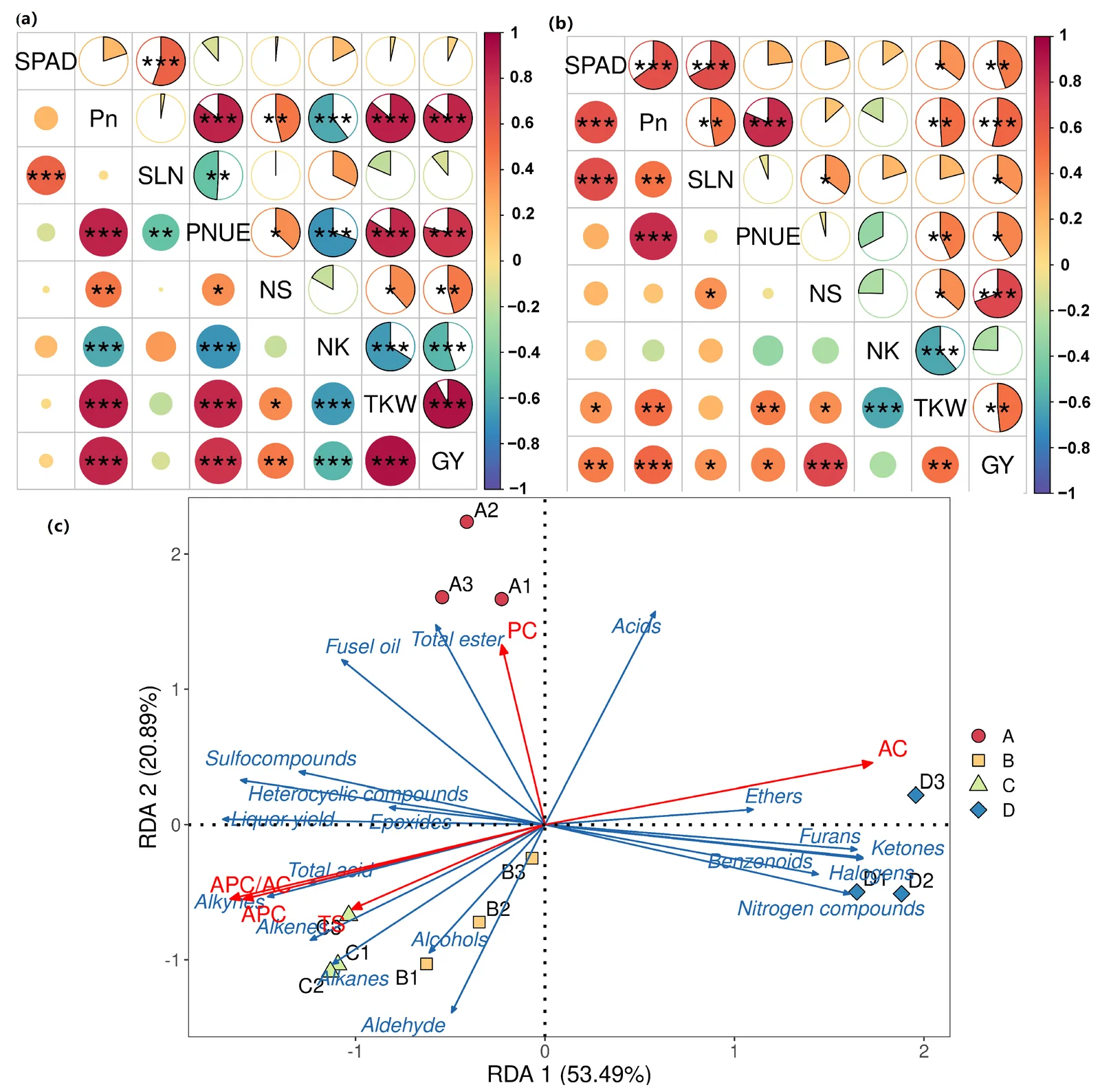
Correlation and redundancy analyses. (**a**,**b**) Correlation analysis between photosynthetic parameters and grain yield at Zhengzhou and Xinxiang sites, respectively. (**c**) Redundancy analysis of grain components and volatile compounds of liquor. The red rays represent starch composition and content. In panels (**a**,**b**), asterisks indicate significance levels: * *p* < 0.05, ** *p* < 0.01, and *** *p* < 0.001.

The correlation analysis between photosynthetic parameters and grain yield revealed that at both Zhengzhou and Xinxiang experimental sites, the Pn of the flag leaf demonstrated significant positive correlations with PNUE, TKW, and grain yield (*p* < 0.001 or *p* < 0.01) (Figure 9). At the Zhengzhou site, PNUE exhibited significant positive correlations with TKW and grain yield at the *p* < 0.001 level. At the Xinxiang site, PNUE displayed significant positive correlations with TKW at *p* < 0.01 and with grain yield at *p* < 0.05.

In the RDA biplot, each point represents a sample, with closer points indicating greater compositional similarity. RDA of grain starch content and liquor-making quality (Figure 9) revealed distinct clustering of samples within treatments and clear separation between treatments, demonstrating significant effects of both N treatment and exogenous melatonin application on liquor-making quality. RDA axis 2 (RDA2) effectively differentiated between HN-T1 (high N + melatonin) and HN-T0 (high N control, no melatonin) treatments, while RDA axis 1 (RDA1) distinguished between MN-T1 (medium N + melatonin) and MN-T0 (medium N control, no melatonin) treatments. The proximity of MN-T1 samples to HN-T0 samples indicates that exogenous melatonin application under reduced N conditions partially compensates for N reduction effects on grain quality. The total starch content, amylopectin content, and amylose-to-amylopectin ratio demonstrated positive correlations with the liquor yield rate, total alcohol content, and total acid content, suggesting that higher total starch and amylopectin contents correlate with elevated total alcohol and acid levels, while amylose exhibited negative correlations with these parameters.

## 4. Discussion

### 4.1. Improved Photosynthetic Parameters and Grain Yield Under Reduced N and Melatonin Application

Crop productivity depends on photosynthetic source capacity. Appropriate N fertilizer management enhances growth, photosynthetic capacity, and crop yield [29]. Researchers have demonstrated that 225 kg∙ha^−1^ N significantly improved Pn, total chlorophyll content, and other physiological traits in winter wheat [30]. Conversely, reduced N supply downregulated genes associated with photosystems I and II, decreasing leaf chlorophyll content and the actual quantum yield of photosystem II photochemistry, ultimately impairing crop yield [31]. In this study, photosynthetic parameters in the flag leaves of both cultivars decreased with reduced N levels. When N application decreased from 225 to 150 kg∙ha^−1^, SPAD of YN901 and Pn of FM8 at Zhengzhou, and PNUE of YN901 and SLN of FM8 at Xinxiang declined significantly. Further reduction to 120 kg∙ha^−1^ significantly decreased most parameters. Melatonin acts as a growth regulator, modulating photosynthesis and protecting the photosynthetic apparatus under stress [32]. It regulates chlorophyll synthesis and degradation by transcription-related genes and hormonal signaling, while protecting photosynthetic proteins and maintaining photosynthetic processes by enhancing photosystem gene transcription, activating antioxidant systems, and promoting the xanthophyll cycle [32,33]. In our study, exogenous melatonin spray improved SPAD, Pn, SLN, and PNUE to varying degrees. Under MN treatment, melatonin application restored Pn and PNUE to levels comparable to those under HN treatment, suggesting that it partially offsets N reduction effects on photosynthetic performance. Correlation analysis revealed highly significant positive relationships between Pn, PNUE, and grain yield. These results demonstrate that exogenous melatonin alleviates N-reduction-induced constraints on photosynthesis, maintaining high photosynthetic capacity and mitigating yield losses under reduced N fertilizer application.

### 4.2. Increased Endogenous Hormones Under Reduced N and Melatonin Application

Exogenous phytohormones and N management can modulate the concentrations of endogenous phytohormones. These hormones function as signaling molecules regulating metabolism and development across plant organs [34,35]. Exogenous melatonin promotes seed germination under stress by regulating ABA/GA balance, reactive oxygen species levels, and metabolic enzyme activity [36]. In this study, the effects of exogenous melatonin on endogenous hormones varied with N levels and developmental stages, demonstrating the close link between hormonal dynamics and grain development. During the late grain-filling stage, exogenous melatonin increased grain IAA and GA_3_ contents, which can extend the duration of rapid grain filling and increase final grain weight [37]. Under LN conditions, ABA accumulation downregulates chlorophyll degradation genes while promoting chlorophyll synthesis [38]. Early researchers demonstrated that elevated ABA concentrations in grains during late filling enhance grain-filling rates and photo-assimilate accumulation [39]. Under reduced N, melatonin application significantly elevated grain ABA content throughout filling, with the largest increase (90.23% higher than the control) occurring at late filling. Thus, melatonin application under N deficit likely maintains high grain-filling activity during late development by promoting ABA, IAA, and GA_3_ accumulation, thereby inhibiting chlorophyll degradation and sustaining grain physiological vitality.

### 4.3. Enhanced Brewing Quality Under Reduced N and Melatonin Application

Starch constitutes the primary component of brewing wheat. The core process of baijiu production involves hydrolyzing wheat starch into glucose, followed by fermentation into alcohol. Thus, the starch content fundamentally determines brewing quality [12]. Liquor yield depends not only on the total starch content but also on the amylose-to-amylopectin ratio. Higher amylopectin content facilitates gelatinization during grain steaming, enhances starch stability post-gelatinization (reducing retrogradation), and improves both liquor yield and quality [40]. However, other researchers have reported that rice varieties with high amylose content yield liquor with significantly elevated levels of 11 volatile flavor compounds (e.g., isoamyl alcohol, isobutanol, ethyl hexanoate) and superior sensory profiles [41]. In this study, both N reduction and exogenous melatonin spray increased total starch and amylopectin contents in the two cultivars. Melatonin application also increased the total ester content of liquor compared with no melatonin spraying. Although melatonin application under HN treatment lowered liquor yield, its combination with MN treatment had no significant effect on liquor yield. An interaction may exist between N fertilizer and exogenous melatonin, and the liquor yield may not only be related to the starch content but also influenced by other grain components. Furthermore, RDA showed that the total starch and amylopectin contents were positively correlated with liquor total acid, total alcohol, and total ester contents.

Protein, wheat’s second-largest nutritional component after starch, critically affects fermentation dynamics, flavor compound formation, and liquor yield [42,43]. However, excessive protein promotes microbial contamination, increases acidity in fermenting grains, disrupts saccharification and lactic acid bacteria fermentation, and imparts harsh off-flavors while hindering starch gelatinization. The acceptable protein content for brewing wheat varies, with the general consensus being 11.0–13.0% [44]. Brewing wheat has lower protein content than non-brewing wheat, typically 12.22–14.47% [45]. At the Xinxiang experimental site, although melatonin application increased the grain protein content, the total protein content remained below 13.0%, particularly under MN conditions where exogenous melatonin spraying resulted in grain protein content of only 10.75–11.45%. The total protein content was high at the Zhengzhou site, possibly because it is highly fertile. Nevertheless, the total protein content was 12.55–14.10%, which is acceptable for brewing materials. Elevated fusel alcohols impair sensory quality, imparting pungency and bitterness while exerting neurotoxic effects [27]. N reduction reduced the fusel alcohol content of brewing liquor, thereby enhancing liquor quality.

Traditional premium baijiu exhibits the most complex aroma profile among distilled spirits worldwide, containing over 1870 identified flavor compounds (e.g., alcohols, esters, acids, aldehydes, pyrazines) [46]. HS-SPME-GC-MS analysis identified main volatile compounds: esters (14.7%), halogens (13.6%), alkanes (13.2%), N compounds (9.5%), alkenes (9.2%), alcohols (8.1%), aromatics (6.2%), and acids (4.4%). Esters predominate in full-aroma baijiu and significantly contribute to its primary aroma characteristics [6,47,48]. Melatonin spray substantially increased total ester content, enhancing overall flavor. Although more volatile compounds decreased than increased under HN-T1 application, certain flavor compounds including 4-methyl-benzeneethanol (floral rose-like), (S)-isopropyl lactate (wine yogurt-like), and butanedioic acid diethyl ester (cooked apple-like) increased, while most alkanes and halogens decreased. Halogen compounds likely have minimal sensory impact [49]. However, under MN-T1 application, more volatile compounds increased rather than decreased, with 1-hexanol and 2-nonanol (green fruity), 3-(methylthio)propanoic acid ethyl ester (pineapple fruity), and allyl methyl sulfide (alliaceous savory) showing higher levels. This suggested that exogenous melatonin at MN levels significantly enriches the liquor’s volatile profile, particularly alcohols, esters, alkenes, aldehydes, N compounds, and sulfur compounds.

## 5. Conclusions

Reduced N application adversely affected flag leaf photosynthetic performance and endogenous hormone levels in wheat grains, resulting in decreased grain yield and alterations in the flavor quality of grain-brewed liquor. Exogenous melatonin application partially mitigated these negative effects by improving photosynthetic performance, PNUE, and endogenous hormonal regulation, thereby enhancing grain yield and favorably modifying the formation of volatile flavor compounds, particularly esters. In detail, MT increased grain yield by 10.61% in YN901 under MN (150 kg·ha^−1^) and by 10.27% in FM8 under LN (120 kg·ha^−1^); total ester content rose by 58.33% under HN and 12.82% under MN. Overall, the combination of moderate N application (150 kg·ha^−1^) and exogenous melatonin effectively balanced reduced N input with grain yield, N use efficiency, and liquor flavor quality. These findings suggest that exogenous melatonin application may provide a promising agronomic strategy for reducing N fertilizer inputs while maintaining wheat productivity and end-use quality, with potential relevance to resource-efficient and sustainable wheat production. Future studies should evaluate the stability and economic feasibility of this strategy across multiple growing seasons, environments, and wheat cultivars, and further optimize melatonin concentration, application timing, and frequency for field-scale implementation.

## Figures and Tables

**Table 1:** Effects of exogenous melatonin application on grain yield and its component factors of brewing wheat grown under reduced nitrogen (N) fertilizer application.

Location	Cultivar	Treatment		Spike Number/ (10^4^ ha^−1^)	Kernel per Spike	1000-Kernel Weight/g	Yield/ (kg ha^−1^)
Zhengzhou	YN901	HN	T1	549.31 ab	53.71 b	35.10 b	8802.35 ab
			T0	556.25 a	50.13 c	32.54 d	7712.66 c
		MN	T1	529.17 bc	54.40 a	36.77 a	8997.19 a
			T0	523.61 c	53.33 b	33.63 c	7982.25 c
		LN	T1	470.14 d	47.71 c	34.10 c	6501.45 d
			T0	471.53 d	47.87 c	33.81 c	6486.89 d
	FM8	HN	T1	543.06 a	45.00 a	39.24 ab	8150.95 a
			T0	551.05 a	42.54 b	38.90 bc	7750.98 a
		MN	T1	560.42 a	43.00 b	38.73 c	7933.20 a
			T0	552.08 a	42.95 b	38.34 d	7727.45 a
		LN	T1	534.38 a	42.92 b	39.57 a	7714.27 a
			T0	506.25 a	43.33 b	38.44 c	7167.30 a
		ANOVA		*F*-value			
		N		3.94 *	69.92 ***	4.26 *	10.59 **
		T		0.01	54.25 ***	104.88 ***	57.87 ***
		C		8.78 **	1202.04 ***	1416.17 ***	507.71 ***
		N×T		0.07	31.96 ***	8.91 **	6.45 **
		T×C		0.03	1.79	36.72 ***	0.99
		C×N		5.56 *	69.03 ***	26.62 ***	27.62 ***
		N×T×C		0.01	10.04 **	17.01 ***	17.15 ***
Xinxiang	YN901	HN	T1	470.14 a	45.52 b	39.29 bc	7147.11 b
			T0	472.92 a	44.99 c	38.97 c	7047.79 b
		MN	T1	468.75 a	48.17 a	40.52 a	7776.89 a
			T0	471.53 a	45.32 b	39.54 b	7182.16 b
		LN	T1	445.14 b	45.75 b	39.18 bc	6782.21 c
			T0	450.00 b	45.20 b	38.98 bc	6739.25 c
	FM8	HN	T1	521.53 a	44.88 a	43.19 c	8592.79 a
			T0	507.64 a	41.10 b	42.60 d	7554.86 b
		MN	T1	464.58 b	42.67 b	45.07 a	7594.33 b
			T0	467.36 b	41.20 b	44.46 b	7275.81 b
		LN	T1	478.47 b	41.75 b	44.28 b	7518.61 b
			T0	474.31 b	37.27 c	44.24 b	6647.45 c
		ANOVA		*F*-value			
		N		59.15 ***	18.68 ***	105.00 ***	170.65 ***
		T		0.01	58.00 ***	36.78 ***	232.66 ***
		C		84.21 ***	230.70 ***	3201.83 ***	293.42 ***
		N×T		1.12	3.29	6.01 **	7.44 **
		T×C		3.71	32.89 **	1.01	0.54
		C×N		31.36 ***	31.78 ***	22.18 ***	2.22
		N×T×C		0.98	16.12 ***	0.53	2.97

The data within the same cultivar followed by different lowercase letters represent a significant difference at 0.05 level. *, **, and *** represent *p* < 0.05, *p* < 0.01, and *p* < 0.001, respectively.

**Table 2:** Effects of exogenous melatonin application on grain protein and starch contents of brewing wheat grown under reduced N fertilizer application.

Location	Cultivar	Treatment		Total Starch Content/%	Amylose Content/%	Amylopectin Content/%	Amylopectin /Amylose	Protein Content/%
Zhengzhou	YN901	HN	T1	70.18 b	19.03 b	51.14 c	2.69 c	13.50 a
			T0	68.28 c	17.50 d	50.79 c	2.90 b	13.55 a
		MN	T1	70.79 a	18.59 c	52.20 b	2.81 b	13.30 a
			T0	70.61 ab	19.49 a	51.12 c	2.62 c	13.20 a
		LN	T1	70.81 a	18.56 c	52.25 b	2.82 b	12.55 b
			T0	70.37 ab	17.06 e	53.31 a	3.12 a	12.65 b
	FM8	HN	T1	71.51 c	16.49 b	55.01 a	3.34 a	14.10 a
			T0	70.33 e	16.39 b	53.94 b	3.29 a	14.10 a
		MN	T1	72.45 b	18.63 a	53.82 b	2.89 b	14.10 a
			T0	70.76 d	18.31 a	52.45 c	2.86 b	13.95 a
		LN	T1	72.77 ab	18.14 a	54.62 ab	3.01 b	13.50 b
			T0	72.89 a	18.20 a	54.69 ab	3.01 b	13.55 b
		ANOVA		*F*-value				
		N		174.08 ***	54.87 ***	54.90 ***	35.56 ***	79.89 ***
		T		144.92 ***	10.99 **	21.20 **	1.42	0.03
		C		489.33 ***	32.53 ***	410.39 ***	73.76 ***	223.81 ***
		N×T		30.03 ***	11.74 **	25.83 ***	9.56 **	1.41
		N×C		27.98 ***	30.09 ***	34.57 ***	28.52 ***	4.00 *
		T×C		0.28	8.79 *	10.96 **	8.15 *	0.24
		N×T×C		24.24 ***	13.86 **	0.29	6.21 *	0.01
Xinxiang	YN901	HN	T1	72.39 b	18.32 b	54.07 c	2.95 d	12.00 a
			T0	72.13 f	17.92 b	54.20 c	3.02 c	11.70 b
		MN	T1	73.57 a	16.69 d	56.88 a	3.41 a	10.75 c
			T0	72.26 e	19.77 a	52.49 d	2.65 e	11.05 d
		LN	T1	73.22 c	18.03 b	55.19 b	3.06 c	11.55 c
			T0	72.82 d	17.39 c	55.43 b	3.19 b	10.65 e
	FM8	HN	T1	71.92 d	17.20 b	54.72 c	3.18 c	12.15 a
			T0	71.25 e	15.81 c	55.44 c	3.51 b	11.90 b
		MN	T1	72.12 c	15.80 c	56.32 b	3.56 b	11.45 e
			T0	72.15 c	19.05 a	53.10 d	2.79 d	10.85 f
		LN	T1	73.43 a	16.48 c	56.95 b	3.46 b	11.70 c
			T0	72.35 b	12.90 d	59.45 a	4.61 a	11.10 d
		ANOVA		*F*-value				
		N		1838.08 ***	418.98 ***	867.19 ***	754.43 ***	619.88 ***
		T		1941.46 ***	1.07	193.70 ***	5.80 *	315.57 ***
		C		1434.98 ***	1566.69 ***	812.51 ***	1978.20 ***	120.14 ***
		N×T		29.83 ***	1057.58 ***	1054.47 ***	1364.86 ***	68.71 ***
		N×C		208.07 ***	160.51 ***	283.58 ***	402.78 ***	2.71
		T×C		8.86 *	181.29 ***	205.49 ***	369.81 ***	17.29 **
		N×T×C		511.88 ***	80.56 ***	27.27 ***	186.25 ***	68.71 ***

The data within the same cultivar followed by different lowercase letters represent a significant difference at 0.05 level. *, **, and *** represent *p* < 0.05, *p* < 0.01, and *p* < 0.001, respectively.

## Data Availability

The original contributions presented in this study are included in the article/Supplementary Material. Further inquiries can be directed to the corresponding author.

## Supplementary Materials

The following supporting information can be downloaded at: https://www.mdpi.com/article/10.3390/foods15183335/s1, Table S1: The volatile substances identified in this study; Table S2: Differentially expressed volatile substances between HN-T0 and MN-T0; Table S3: Differentially expressed volatile substances between HN-T1 and HN-T0; Table S4: Differentially expressed volatile substances between MN-T1 and MN-T0. Figure S1. Monthly mean temperature and precipitation in Zhengzhou and Xinxiang during the wheat growing season.
